# Fostering effective hybrid human-LLM reasoning and decision making

**DOI:** 10.3389/frai.2024.1464690

**Published:** 2025-01-08

**Authors:** Andrea Passerini, Aryo Gema, Pasquale Minervini, Burcu Sayin, Katya Tentori

**Affiliations:** ^1^Department of Information Engineering and Computer Science, University of Trento, Trento, Italy; ^2^School of Informatics, University of Edinburgh, Edinburgh, United Kingdom; ^3^Center for Mind/Brain Sciences, University of Trento, Trento, Italy

**Keywords:** hybrid intelligence, human-AI collaboration, LLMs, biases, mutual understanding, complementary team performance

## Abstract

The impressive performance of modern Large Language Models (LLMs) across a wide range of tasks, along with their often non-trivial errors, has garnered unprecedented attention regarding the potential of AI and its impact on everyday life. While considerable effort has been and continues to be dedicated to overcoming the limitations of current models, the potentials and risks of human-LLM collaboration remain largely underexplored. In this perspective, we argue that enhancing the focus on human-LLM interaction should be a primary target for future LLM research. Specifically, we will briefly examine some of the biases that may hinder effective collaboration between humans and machines, explore potential solutions, and discuss two broader goals—mutual understanding and complementary team performance—that, in our view, future research should address to enhance effective human-LLM reasoning and decision-making.

## 1 Introduction

The release of chatGPT has raised unprecedented attention and generated high expectations on the capabilities of AI systems that leverage large language model (LLM) technologies. These systems have demonstrated impressive results across a wide range of tasks (Liu et al., 2023b; Yang et al., 2024), such as language translation (Jiao et al., 2023), text summarization (Pu and Demberg, 2023), question-answering (Bahak et al., 2023), reasoning (Bang et al., 2023), and text generation (Chen et al., 2023b; Jeblick et al., 2022), prompting questions about the potential emergence of “thinking machines” and artificial general intelligence sparks (Bubeck et al., 2023). However, several studies have highlighted the limitations of these systems. Just to provide a few examples, they have been shown to provide entirely fabricated information (Huang et al., 2023), to exhibit sensitivity to small changes in the way questions are posed (Pezeshkpour and Hruschka, 2023), and to agree with human opinions regardless of content (Sharma et al., 2023).

Human beings are also far from being entirely rational, and not in an obvious way. The deviations of human reasoning from normative benchmarks create an intriguing puzzle that is not yet completely understood in Cognitive Science. On the one hand, systematic and persistent biases manifest even in well-motivated and expert individuals engaged in simple, high-stakes probability tasks (Baron, 2023). This suggests that reasoning errors do not stem from carelessness, computational limitations, or lack of education, nor are they necessarily caused by “external constraints” such as inadequate information or time pressure. On the other hand, individuals are often capable of complex inferences (Tenenbaum et al., 2011; Mastropasqua et al., 2010). In particular, evidential reasoning—i.e., the assessments of the perceived impact of evidence—appears to be quite effective, demonstrating greater accuracy and consistency over time compared to corresponding posterior probability judgments (Tentori et al., 2016). This holds true even though, from a formal standpoint, calculating the former is no easier than calculating the latter.

Notably, there are solid reasons to believe that neither LLM-based AI systems nor humans will turn into completely rational agents anytime soon. With regard to the former, the inherent mechanisms of LLMs impose significant constraints on their capabilities. Bender and Koller (2020) and Bender et al. (2021) coined the term “stochastic parrots” to highlight the fact that LLMs focus on form over meaning and stressed the difficulty of getting the latter from the former. More recently, Mahowald et al. (2024) formalized the problem in terms of the distinction between formal and functional linguistic competence, arguing that LLM architectures require substantial modifications to have a chance of achieving the latter. Finally, Xu et al. (2024b) indicated that inconsistencies between LLMs and the real world are, to some extent, inevitable. In a similar vein, efforts to enhance human rationality by using visual aids (Khan et al., 2015), promoting accountability (e.g., Boissin et al., 2023), or shaping external environments (e.g., *nudging*, Thaler and Sunstein, 2009) have often yielded modest results that are not easily generalizable to other contexts (Chater and Loewenstein, 2023). The limited effectiveness of these interventions suggests that the causes of reasoning biases are deeply ingrained in our cognitive processes, and we cannot expect to eradicate them, at least not in the near future.

Humans and LLMs are not only imperfect yet highly capable, but they also differ significantly in their respective strengths and weaknesses (Chang et al., 2023; Shen et al., 2023; Felin and Holweg, 2024; Leivada et al., 2024). Thus, while mere interaction between the two does not guarantee success, a carefully designed human-LLM synergy has the potential to prevent critical problems and achieve results that surpass what either could accomplish alone. Indeed, recent research highlights human-LLM collaboration as a key direction toward realizing genuinely human-centered AI. (Dellermann et al., 2019; Akata et al., 2020; Lawrence, 2024; Wang et al., 2024; Ma et al., 2024; Liao and Wortman Vaughan, 2024). However, in our view, effectively addressing this issue necessitates a significant shift in perspective. The primary challenge we must confront—and one that will increasingly be faced in the future—lies not so much in the specific boundaries of human rationality or the current technological limitation of LLMs, but rather in the nature and severity of biases that can arise from their *interaction*. For this reason, we do not aim to provide an exhaustive list of the many cognitive biases that individuals—and, in some cases, LLMs—exhibit. Instead, we will focus on three major problems of LLMs—*hallucinations, inconsistencies* and *sycophancy*—demonstrating how they can impact the interplay with humans. We will then discuss two key desiderata, *mutual understanding* and *complementary team performance*, which, in our opinion, future research should address more comprehensively to foster effective human-LLM reasoning and decision-making.

## 2 Potential weaknesses in human-LLM interaction

One of the most well-known problems of LLMs is hallucination, which refers to their distinct possibility of generating outputs that do not align with factual reality or the input context (Huang et al., 2023). Hallucinations in LLMs have several causes, from flawed data sources (Lin et al., 2022b) to architectural biases (Li et al., 2023b; Liu et al., 2023a). To exacerbate the issue, when LLMs engage in hallucination, they maintain an aura of authority and credibility by generating responses that appear coherent and well-formed in terms of natural language structure (Berberette et al., 2024; Su et al., 2024). Such a behavior can easily lead to an *automation bias* (Cummings, 2012), where users tend to over-rely on information and suggestions from automated systems compared to those from their peers. Indeed, while people can easily detect nonsensical or blatantly unrelated outputs from LLMs when they have a good knowledge of the topic, they are more likely to overlook such errors when they lack expertise in the subject. This creates a paradox: one must already possess the correct answer to reliably avoid being misled by LLMs. Nonetheless, expertise itself is not a guarantee that everything will go smoothly. Humans, including professionals such as, for example, physicians, often exhibit a tendency known as *overconfidence* (Hoffrage, 2022), where they tend to overestimate their abilities or the accuracy of their knowledge. Predicting which of these somewhat opposite attitudes would prevail in a given interaction between humans and LLMs is extremely difficult. LLMs could, in principle, counteract overconfidence by providing negative feedback to users. However, what might seem like an easy solution runs into another characteristic of these systems: their tendency toward sycophancy, which is the inclination to please users by generating responses that are agreeable rather than strictly accurate, especially when trained with biased human feedback or tasked with generating content in subjective domains (Sharma et al., 2023; Ranaldi and Pucci, 2023; Wei et al., 2023). Furthermore, overly critical feedback may lead to *algorithm aversion bias* (Dietvorst et al., 2014), where users disregard information that conflicts with their previous beliefs, even when it is actually pertinent and correct. This bias reflects the skepticism with which humans—especially professionals in high-stakes fields like healthcare and law, where accountability is paramount—often view the advanced capabilities of LLMs (Park et al., 2023; Cheong et al., 2024; Choudhury and Chaudhry, 2024; Eigner and Händler, 2024; Watters and Lemanski, 2023). Additionally, algorithm aversion may be fueled by a loss of confidence following unsatisfactory initial interactions (Huang et al., 2024; McGrath et al., 2024). In particular, LLM inconsistency—reflected in their tendency to produce varying outputs for very similar (or even identical) inputs—can easily leave lasting impressions of unreliability. This issue is exacerbated by high prompt sensitivity, where the LLM tend to provide different answers even with slight changes in how questions are phrased (Pezeshkpour and Hruschka, 2023; Voronov et al., 2024; Mao et al., 2024; Sayin et al., 2024). As a consequence, individuals may become increasingly reluctant to utilize LLMs when confronted with important reasoning and decision-making tasks.

Let us now consider a situation that, in principle, would be expected to unfold more smoothly—namely, one in which neither LLMs nor humans are outright incorrect. It might be assumed that accuracy alone would suffice to prevent errors; however, unfortunately, this is not necessarily the case. A well-known bias that could persist or even intensify in interactions where humans feel competent and LLMs provide reliable evidence is *confirmation bias*: the tendency to selectively seek, interpret, and recall information that supports existing beliefs (Nickerson, 1998). Indeed, when users query LLMs based on initial hypotheses and the models provide selective answers mainly based on local context, a vicious cycle can be fueled. A closely related cognitive bias that may similarly be exacerbated in interactions with LLMs is *belief bias*, that is the tendency to conflate the validity of an argument with the confidence placed in its conclusion (Evans et al., 1983). For instance, users might fail to realize that evidence obtained too easily, thanks to the “efficiency” of LLMs in supporting a cherished hypothesis, is not as comprehensive or conclusive with respect to the hypothesis in question as it may seem. Another risk is *overestimating redundant information*: without full control over the sources LLMs draw from, users may overlook redundancy and mistakenly believe they are gaining new evidence to support a particular belief or prediction, when in fact they are not (Bohren, 2016). Similarly, interactions between individuals and LLMs might be susceptible to the so-called *anchoring* to initial hypotheses or inquiries (Tversky and Kahneman, 1974), as well as to *order effects* (Hogarth and Einhorn, 1992). These biases refer, respectively, to the tendency to rely excessively on reference points (even if irrelevant) when making estimates, and to assign greater importance to, or better recall, the first or last pieces of information encountered, at the expense of less available content.

Ex-post evaluation of interactions between human reasoners and LLMs (i.e., the assessment of their interactions after they have taken place) is not immune to errors either. Among the major issues, one cannot help but consider the well-known *hindsight bias*, which is the tendency to perceive events, once they have occurred, as more predictable than they actually were (Arkes, 2013). For instance, individuals might overestimate the accuracy of LLM predictions simply because they overlook how often the original outputs of these models are tentative and inconclusive. Similarly, due to the selective information provided by the models, individuals may underestimate their own initial uncertainties. The concern is that if this misinterpretation of the interaction occurs collaboratively, biases like the one discussed above could be reinforced rather than mitigated.

In conclusion, interactions between the LLM and the user can amplify their inherent weaknesses or even create new ones. This underscores the urgent need for methodological innovations that integrate LLM behaviors with new, interactively designed solutions; without this, they may fail or even backfire.

## 3 Toward effective human-LLM interaction

In this section, we will first present potential solutions to three major challenges of LLMs: hallucinations, inconsistencies, and sycophancy. We will then discuss how fostering mutual understanding and enhancing complementary team performance are crucial for achieving effective collaboration in reasoning and decision-making between humans and LLMs.

### 3.1 Detecting and mitigating the impact of hallucinations

Hallucinations are extensively studied in the field of Natural Language Processing (NLP), with various approaches proposed to prevent, detect, or mitigate their occurrence (Huang et al., 2023; Ji et al., 2023a; Rawte et al., 2023; Zhang et al., 2023). Following Huang et al. (2023), we categorize hallucinations into *factuality hallucinations*, where the model generates responses that contradict real-world facts, and *faithfulness hallucinations*, where the model's responses are not aligned with user instructions or the provided context. The latter can be further divided into *intrinsic hallucinations*, involving responses that directly contradict the context, and *extrinsic hallucinations*, in which the generated content cannot be verified or refuted based on the context (Maynez et al., 2020).

One way to improve the factuality of model-generated content is via *retrieval augmented generation* (Lewis et al., 2020), which conditions the generation process on documents retrieved from a corpus such as Wikipedia or Pubmed (Shuster et al., 2021; Xiong et al., 2024; Zakka et al., 2024). However, LLMs can still disregard provided information and rely on their parametric knowledge due to intrinsic mechanisms (Jin et al., 2024; Xu et al., 2024a) or sensitivity to prompts (Liu et al., 2024). Another solution is adapting the generation process—referred to as *decoding*—to produce more factual responses (Lee et al., 2022; Burns et al., 2023; Moschella et al., 2023; Li et al., 2023a; Chuang et al., 2023), and post-editing to refine the originally generated content, leveraging the self-correction capabilities of LLMs (Dhuliawala et al., 2023; Ji et al., 2023b). Decoding can also be adapted to generate outputs that are more faithful to the user instructions or the provided context.

Recent efforts to mitigate faithfulness hallucinations focus on two main areas: *context consistency*, which aims to improve the alignment of model-generated responses with user instructions and the provided context (Tian et al., 2019; van der Poel et al., 2022; Wan et al., 2023; Shi et al., 2023; Gema et al., 2024; Zhao et al., 2024b); and *logical consistency*, which seeks to ensure logically coherent responses in multi-step reasoning tasks (Wang et al., 2023a). Decoding-based methods can be coupled with *post-hoc* hallucination detection approaches (Manakul et al., 2023; Min et al., 2023; Mishra et al., 2024) to define a reward model and adaptively increase the likelihood of hallucination-free generations (Wan et al., 2023; Amini et al., 2024; Lu et al., 2022, 2023; Deng and Raffel, 2023). From the user's perspective, a crucial factor in reducing LLM hallucinations is ensuring that queries are well-constructed, unambiguous, and as specific as possible, since vague or poorly phrased prompts can increase the likelihood of hallucinations (Watson and Cho, 2024).

Although the solutions discussed above can help reduce hallucinations, they will remain, to some extent, inevitable due to the complexity of the world that LLMs attempt to capture (Xu et al., 2024b). A complementary approach is to enhance humans' awareness in managing such occurrences by enabling LLMs to provide uncertainty estimates alongside their outputs. The approaches implemented so far in this line of research fall into three categories (Xiong et al., 2024): *logit-based estimation, verbalization-based estimation*, and *consistency-based estimation*. Logit-based estimation requires access to the model logits and typically measures uncertainty by calculating token-level probability or entropy (Guo et al., 2017; Kuhn et al., 2023). Verbalize-based estimation works by directly requesting LLMs to express their uncertainty via prompting strategy (Mielke et al., 2022; Lin et al., 2022a; Xiong et al., 2024; Kadavath et al., 2022). Finally, consistency-based estimation works under the assumption that the most consistent response signifies the least hallucination in the LLM generations (Lin et al., 2023; Chen and Mueller, 2023; Wang et al., 2023b; Zhao et al., 2023). Additionally, recent studies are exploring a new and promising strategy in which LLMs learn to generate citations (Gao et al., 2023; Huang and Chang, 2023). In this way, users can assess the reliability of the outputs provided by LLMs by examining, and potentially directly accessing, their sources.

### 3.2 Improving robustness

Variability, prompt brittleness, and inconsistencies in LLM outputs across different conditions, domains, and tasks (Gupta et al., 2023; Zhou et al., 2024; Tytarenko and Amin, 2024) pose significant challenges for ensuring effective interaction with humans and can substantially exacerbate their algorithmic aversion. Efforts to enhance the robustness of LLMs have included adjustments during training, as well as *post-hoc* solutions applied after learning has taken place. Regarding the former, recent research has increasingly recognized the value of including domain experts within development teams (e.g., Med-Gemini in healthcare; Saab et al., 2024, FinMA in finance; Xie et al., 2023, and SaulLM; Colombo et al., 2024). Post-training techniques aimed at mitigating prompt sensitivity while preserving performance include *in-context learning adjustments* (Gupta et al., 2023), *task-specific context attribution* (Tytarenko and Amin, 2024), and *batch calibration* (Zhou et al., 2024).

Among the solutions for enhancing LLM robustness are those that directly involve humans, within both perspectives mentioned above. Zhao et al. (2024c) introduced *consistency alignment training* to better align LLM responses with human expectations, fine-tuning LLMs to provide consistent answers to paraphrased instructions Post-training methods involving humans often focus on improving in-context learning examples to be given to the LLMs, by coupling input-output pairs with their corresponding human-generated natural language explanations (He et al., 2024).

Another approach to increasing robustness involves introducing an intermediate step between the user and the model, known as *guardrailing* (Inan et al., 2023; Rebedea et al., 2023), which literally means 'keeping the model on track.' This step evaluates the input and/or output of LLMs to determine if and how certain enforcement actions should be implemented. Common instances include refraining from providing answers that could lead to misuse or blocking responses that contain harmful, inappropriate, or biased content.

### 3.3 Dealing with sycophancy

Sycophancy is a sort of ‘side effect' of the attempt to maximize user satisfaction and the training of LLMs on datasets that include texts generated by humans, where interlocutors often seek to meet each other's expectations. This issue with current LLMs is, of course, not independent of other limitations, but they can exacerbate one another. Indeed, LLMs often hallucinate and become inconsistent in order to appease user prompts, especially when these are misleading. By compelling LLMs not to accommodate these prompts, it could thus lead to a reduction of multiple limitations. On this line, Rrv et al. (2024) showed how popular hallucination mitigation strategies can be effectively used also to reduce the sycophantic behavior of LLMs in factual statement generation.

Other solutions to address sycophancy involve fine-tuning LLMs over aggregated preferences of multiple humans (Sharma et al., 2023), generating synthetic fine-tuning data to change model behavior (Wei et al., 2023) or applying activation editing to steer the internal representations of LLMs toward a less sycophantic direction (Panickssery et al., 2024). To preserve the original capabilities of the LLM as much as possible, Chen et al. (2024) propose *supervised pinpoint tuning*, where fine-tuning is confined to specific LLM modules identified as responsible for the sycophantic behavior.

Finally, Cai et al. (2024) proposed a shift in perspective, termed *antagonistic AI*, a provocative counter-narrative to the prevailing trend of designing AI systems to be agreeable and subservient. According to this approach, human-LLM interactions could benefit from confrontational LLMs that challenge users, even to the point of being blunt if necessary. More specifically, the authors argue that forcing users to confront their own assumptions would, at least in certain situations, promote critical thinking. This intriguing proposal has yet to be implemented or undergo empirical testing. Complementary to this, Tessler et al. (2024) demonstrated that LLMs can assist humans in finding common ground during democratic deliberation by facilitating effective perspective-taking among group members. We believe these approaches could indeed help people identify potential pitfalls in their reasoning and decision-making processes if complemented by cognition-aware interaction strategies to avoid exacerbating algorithmic aversion bias.

### 3.4 Fostering mutual understanding

The blossoming area of Explainable AI (XAI; Miller, 2018; Gunning et al., 2019; Longo et al., 2024) aims at addressing the problem of explaining the outputs of black-box models to humans, focusing either on single predictions (*local explainability*) or the entire model (*global explainability*). *Explanatory interactive learning* (Teso and Kersting, 2019) builds upon XAI approaches to allow humans to guide machines in learning meaningful predictive patterns while avoiding confounders and shortcuts. However, XAI faces several challenges, from the lack of faithfulness in the generated explanations (Camburu et al., 2019) to the impact of human cognitive biases on evaluating these explanations (Bertrand et al., 2022), including the risk of increasing automation bias (Bansal et al., 2021; Buçinca et al., 2021). The opposite direction—helping machines to understand humans—is equally challenging. Eliciting human knowledge proves inherently difficult, as it is often implicit, incomplete, or incorrect (Patel et al., 1999). Expert judgments, although intuitive, depend on rich mental models that manage incomplete or conflicting information, complicating the representation of this knowledge for machine learning models (Klein et al., 2017; Militello and Anders, 2019).

Compared to other black-box models, LLM-based architectures offer both advantages and disadvantages in terms of mutual understanding. A clear advantage is their use of natural language for communication, enabling conversational sessions where human feedback is integrated into subsequent interactions. However, this natural mode of interaction can be misleading for human partners. Indeed, empirical studies show that users increase their trust in LLM responses when these are accompanied by explanations, even if the responses are deceptive (Sharma et al., 2024). Although various attempts to foster human-LLM alignment through training and interaction strategies have been made (Wang et al., 2023c), LLMs still represent concepts through distributional semantics (Lenci and Sahlgren, 2023), which differs significantly from human semantic understanding (Bender and Koller, 2020). One consequence is that, like many other sub-symbolic machine learning models, LLMs are prone to shortcut learning (Du et al., 2023), a tendency to rely on non-robust features that are spuriously correlated with ground-truth supervision in the training data, yet fail to generalize in out-of-distribution scenarios. XAI approaches are starting to shed light on the reasoning mechanisms of LLMs (Zhao et al., 2024a), but further research is needed for them to produce reliable proxies of the trustworthiness of LLM outputs.

Finally, effective interaction between humans and LLMs requires a form of mutual understanding that involves a theory of mind (ToM; Premack and Woodruff, 1978)—the ability to infer what others are thinking and how this differs from our own thoughts, a crucial precondition for effective communication and cooperation. Recent studies (van Duijn et al., 2023; Kosinski, 2024; Strachan et al., 2024) have shown that larger LLMs, such as GPT-4, made significant progress in ToM, performing on par with, and sometimes even surpassing, humans under certain conditions. However, this competence primarily reflects an ability to simulate human-like responses rather than a genuine mastery of the cognitive processes involved in ToM reasoning. Achieving authentic ToM in LLMs will require further advancements, such as leveraging external memory systems (Li and Qiu, 2023; Schuurmans, 2023) and, eventually, developing machine metacognition (Johnson et al., 2024).

### 3.5 Targeting complementary team performance

Machine learning methods are typically evaluated in terms of their performance as standalone entities. LLMs are no exceptions to this rule and most research focuses on improving their performance over pre-defined benchmarks (Hendrycks et al., 2021; Liang et al., 2022; Petroni et al., 2021; Chiang et al., 2024). A recent trend has started to question this perspective, advocating for explicit inclusion of the human component in the development and use of these systems (Donahue et al., 2022; Hemmer et al., 2021; Guszcza et al., 2022; Sayin et al., 2023). The notion of *complementary team performance* (CTP; Bansal et al., 2021) has been introduced to evaluate whether team accuracy is higher than either the human or the AI working alone (Hemmer et al., 2021, 2024; Campero et al., 2022). Quite interestingly, studies have shown that human-AI teams can outperform humans but often do not exceed the performance of AI alone (Bansal et al., 2021; Hemmer et al., 2021), highlighting the complexity of achieving good CTP in practice.

Within the machine learning community, researchers have developed *ad hoc* learning strategies to improve CTP. The most popular is *selective classification* (Geifman and El-Yaniv, 2017), where the machine selectively abstains from providing predictions it deems too uncertain. Several selective classification strategies have been proposed in the NLP community, especially in question-answering tasks (Xin et al., 2021; Varshney et al., 2022). A limitation of selective classification is that it does not take into account the characteristics of the person to whom the prediction is deferred. *Learning to defer* (Madras et al., 2018) is an advancement over selective classification, in which human expertise is being modeled and accounted for in choosing when to abstain. *Learning to complement* (Wilder et al., 2021) further extends this line of research by designing a training strategy that directly optimizes team performance. The next challenging yet crucial step will be to adapt these strategies to handle arbitrary users and general-purpose human-LLM reasoning and decision-making tasks.

A major limitation of current solutions for learning to defer/complement is that they rely on a *separation of responsibilities* between the human and the machine. Banerjee et al. (2024) argued that this is suboptimal because it leaves humans completely unassisted in the (presumably difficult) cases where the machine defers, while fostering their automation bias when the machine does not defer. The authors proposed an alternative strategy, *learning to guide*, in which the machine is trained to provide helpful hints to assist the user in making the right decision.

Other promising research directions include adapting strategies that have been developed and proven effective in other areas of AI to LLMs. Among these is *conformal prediction* (Angelopoulos and Bates, 2023), which allows a model to return prediction sets that, according to a user-specified probability, are guaranteed to contain the ground truth. This has been empirically shown to improve human decision-making (Straitouri et al., 2023; Cresswell et al., 2024), and it is beginning to be extended to LLM architectures [*conformal language modeling* (Quach et al., 2024)]. Another approach is *mixed-initiative interaction* (Allen et al., 1999; Barnes et al., 2015), where each agent contributes its strengths to the task, with its level of engagement dynamically adjusted to the specific issue at hand. Recent studies have introduced methods for formalizing prompt construction to enable controllable mixed-initiative dialogue generation (Chen et al., 2023a). Finally, *argumentative decision making* (Amgoud and Prade, 2009) applies argumentation theory to enhance team performance by structuring interactions as sequences of arguments and counter-arguments. Recently, argumentative LLMs (Freedman et al., 2024) have been proposed and tested as a method using LLMs to construct formal argumentation frameworks that support reasoning in decision-making.

## 4 Conclusion

A human-centered approach to AI has been increasingly promoted by governmental institutions (European Commission, 2020), with legal requirements in many countries mandating human oversight for high-stakes applications (Government of Canada, 2019; European Commission, 2021). Building on this perspective, we have discussed a range of strategies through which the main limitations of current LLMs could be addressed and proposed two fundamental desiderata—mutual understanding and complementary team performance—that, in our view, should guide future research on LLMs and beyond. Indeed, while this manuscript focuses on LLMs due to their widespread adoption, including among lay users, many of the points raised may well apply to multimodal and general-purpose foundation models (Sun et al., 2024) when interacting with humans.

The advocated shift in perspective would require greater involvement of cognitive scientists in shaping approaches to overcome LLM limitations and assess their effectiveness, significantly altering priorities regarding problems and goals for the success of LLMs. Future work could explore new evaluation metrics inspired by cognitive science to better measure the effectiveness of these approaches. Indeed, only by combining the knowledge and exploiting the strengths of both humans and LLMs can we have a real chance to achieve a true partnership—one that is not only more effective in reducing human-machine biases but also more transparent and fair.

## Data Availability

The original contributions presented in the study are included in the article/supplementary material, further inquiries can be directed to the corresponding author.
